# An evaluation of a peer supervision pilot project among community health workers in rural Uganda

**DOI:** 10.4314/ahs.v22i2.74

**Published:** 2022-06

**Authors:** Grace Nakibaala, Agnes Watsemba, Brian Ssali, Frank Namugera, Phionah Katushabe, Maggie Carleen, Molly Christiansen, Emilie Chambert

**Affiliations:** 1 Living Goods, Uganda; 2 Living Goods Global office

**Keywords:** Community health workers, peer supervision, supervision, Uganda

## Abstract

**Background:**

Living Goods operates a Community Health Worker (CHW) program in 19 districts of Uganda, where CHWs are supervised by full time Community Health Supervisors. This model is effective, but expensive. Evidence indicates that peer supervision can be a substitute and cheaper model for CHW supervision. We describe our experience and outcomes while implementing peer supervision among CHWs in Mayuge district

**Objectives:**

1. To compare health services delivery outcomes between the two supervision models. 2. To compare costs of the two supervision models..

**Methods:**

Internal organizational records from January to December 2019 were reviewed. Focus group discussions and in-depth interviews with participating CHWs were also conducted. Qualitative analysis was performed using thematic content analysis. Quantitative data was summarized to generate averages, percentages and graphs.

**Findings:**

CHWs under the peer supervision performed better than those under standard supervision against all key performance indicators. The total cost to maintain the peer supervision model for 1 year was USD $176 per CHW versus USD $273 among CHWs under the standard supervision model. Peer supervision thus resulted in overall cost savings of 36%. There was lower attrition among CHWs under peer supervision compared to standard supervision (10% versus 17%).

**Conclusions:**

Peer supervision is a feasible and more affordable model of supervising CHWs.

## Background

The World Health Organization recommends the use of Community Health Workers (CHWs) to address the growing shortage of health workers, particularly in low-income countries^1^. The umbrella term “CHW” embraces a variety of community health aides selected and trained to render certain basic health services to the communities they come from^2^. Extensive research has shown that CHW programs are effective in delivering a range of preventive, promotive, and curative services related to reproductive, maternal, newborn, and child health^3^–^8^. Despite these benefits, many challenges including insufficient supervision, quality control, and support make CHW programs difficult to maintain^9^.

The quality of CHW supervision is often constrained due to lack of skills and tools, time for supervision, travel expenses, and logistics, as well as financial obstacles^9^,^10^–^13^. According to Tulenko^13^, CHWs have special supervision needs because their level of education and literacy is usually lower than other health workers and their period of formal CHW training is often only a few weeks. In addition, they usually practice alone, providing little room for reinforcement or support. In combination, these factors can result in poor quality work, burnout, absenteeism, and attrition. Therefore, investing in high-quality CHW supervision can help CHWs perform better. Evidence at the global level suggests that regular and systematic supervision, with clearly defined objectives, can improve the motivation and performance of CHWs involved in primary health care^14^–^25^. Supervision focused on supportive approaches, quality assurance, and problem solving is generally considered most effective at improving CHW performance^10^,^26^.

Living Goods operates a community health program of more than 4,200 CHWs in 19 districts of Uganda. All CHWs are digitally empowered with a phone and diagnostic Smart Health application, equipped with commodities and supplies, supervised, and compensated. In Living Goods' standard supervision model, 25–30 CHWs are supervised by a Community Health Supervisor (CHS) who is a full-time employee of the organization. The CHS reviews CHWs' performance and carries out field visits with each of the CHWs, coaching and mentoring them to achieve their targets. Whereas this model has proven to be effective, it is expensive to implement. The unit cost of supervising one CHW for 1 year under this model is USD$273. Evidence indicates that peer supervision can be a substitute for standard supervision since it is more cost-efficient, results in stronger commitment to work^10^,^27^–^30^, and leads to CHWs finding more creative solutions to problems^29^.

**Figure 1:**
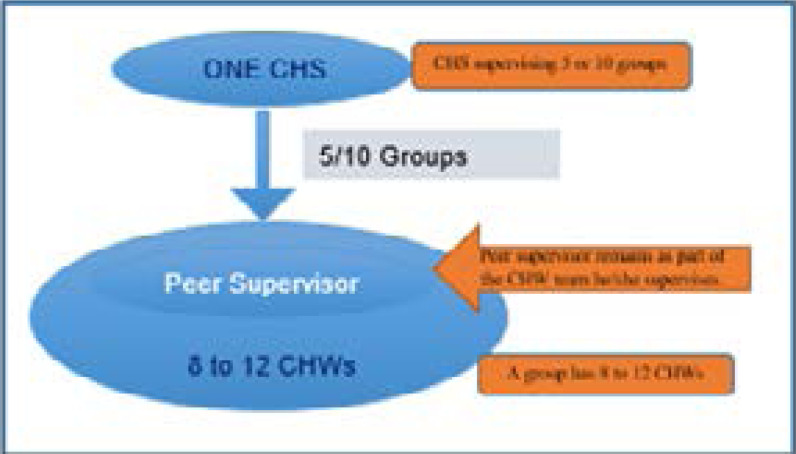
Peer supervision model

Peer supervision is an approach in which selected CHWs take on supervisory roles through peer-to-peer learning, support, and problem solving^27^. In November 2018, Living Goods piloted a peer supervision model for its CHWs working within one district. The model borrows best practices from models implemented elsewhere, like the Lady Health Worker Program (Pakistan), Integrated Management of Childhood Illness (Benin), and Health Extension Workers Program (Ethiopia). From the 441 CHWs supported in Mayuge district, 211 (48%) were selected to participate in the peer supervision pilot while the rest of the CHWs were left under the existing supervision approach. Under peer supervision, CHWs in close geographic proximity grouped themselves together into 8 to 12 individuals per group and voted on a respected leader among themselves, termed a Peer Supervisor. A total of 20 Peer Supervisors were selected. The Peer Supervisors were centrally trained on mentorship, coaching, and Android support at the beginning of the pilot. They subsequently attended monthly meetings to reinforce their knowledge, receive updates, and troubleshoot problems. Each Peer Supervisor received USD$5.4 as weekly facilitation to cater for transport and communication costs as they supervised their peers. The groups were provided with a monthly performance-based incentive capped at USD$2.7 per CHW based on pre-set targets on key indicators. The responsibilities of the CHS and the Peer Supervisor are highlighted in Table 1. Between 5 to 10 groups would be overseen by a Living Goods CHS (Figure 1 below). The CHS was expected to visit each group weekly for 2 to 3 hours and to support the Peer Supervisor and the other CHWs to deliver impact, strengthen their capacity, and deliver needed commodities.

**Table 1:** Responsibilities of a CHS and Peer Supervisor during the pilot

Responsibilities of a CHS	Responsibilities breakdown under peer supervision
Motivate CHWs Implement marketing and promotional efforts to support CHWs' sales goals. Lead monthly In-Service meeting of CHWs. Support the management of financial operations. Support the management and maintenance of inventory. Support the management of relations between Living Goods and the implementing partner organizations. Work closely with the district	For the CHS Coach the peer supervisors Review performance and quality of all CHWs Conduct group field visits/meetings Re-stock CHWs Conduct refresher trainings as needed For the Peer supervisor Motivate the CHWs to achieve their targets Review CHW performance CHW stock check Door to door activities/ movement with CHWS

In this paper, we describe our experience implementing the model, report program outcomes from November 2018 to November 2019, and provide qualitative analysis of the successes and challenges of the program.

## Methods

### Study design

A cross-sectional mixed-methods study was conducted. Program records on pre-existing key performance indicators (KPIs) were reviewed and the findings were triangulated with qualitative methods to document Peer Supervisors', CHWs', and CHSs' experiences with peer supervision; to establish the challenges and how they were mitigated during the pilot; and to document lessons learnt and best practices for scale up.

### Setting

The peer supervision model was implemented in Mayuge District, located in the Eastern region of Uganda. The district is bordered by Iganga District to the north, Bugiri District to the northeast, Namayingo District to the east, the Republic of Tanzania to the south, and Jinja District to the west. The 2014 National Census estimated the population of Mayuge District to be 473,239. Living Goods started operations in Mayuge in 2009. Within the district, Living Goods has a presence in 12 (86%) sub-counties, with an estimated coverage of 53% (238) of the villages.

### Data collection

All quantitative program data from January to December 2019 was reviewed, comparing CHW participants in the peer supervision pilot against those within the same district under standard supervision, on a set of predefined KPIs. KPIs included number of sick children assessed and treated, postnatal follow-ups, and pregnancies supported. This data is routinely extracted from the CHW app and exported into R for ongoing analysis and program learning. Whereas all program data for all CHWs was considered in the analysis, a random sample of 29 CHWs was selected to participate in three focus group discussions (FGDs). A fourth FGD was conducted among 11 Peer Supervisors. The sample size was determined based on the principle of theoretical saturation, at which further interviews would not yield any new information^36^. The FGDs were heterogenous, and the power dynamics were managed by skilful and strong moderation to facilitate and manage conflicting perspectives and personalities, while maintaining the session's open and inviting ambience for all participants to feel able to talk openly and honestly. In-depth interviews (IDI) were held with all three CHSs and the branch manager who directly supported the intervention. The interviews were steered by interview guides which explored CHWs' experiences, perceived support, motivation, and benefits of peer supervision. The guides also captured background characteristics of all respondents, including age, sex, education level, number of households and their villages, and the period spent with Living Goods as a CHW. CHW 1-year attrition was also considered by comparing the number of CHWs active at the end of the 12 months versus those at the start of the program. The interviews were facilitated by a moderator who was supported by a note taker. Audio recordings were also made to allow for complete capture of the discussions. Informed consent was sought from all FGD and IDI participants. Costs for both routine supervision and peer supervision were documented throughout the year of study.

### Data analysis

Quantitative data was analysed using R to generate sums, frequencies, and percentages. Graphical presentation of the data was also made to compare CHWs under peer supervision with those who were not. Overall cost data was divided by the number of CHWs in the pilot and then compared with those who were not.

Qualitative data was transcribed and analysed using the thematic analysis approach. A list of themes based on the interview questions was first created. All transcripts were read several times to ensure that their meaning and context were understood. Comparisons of responses across categories, themes, participants, and locations to examine and triangulate the data were completed to see if there were any differences in the understanding of different responses to the evaluation questions.

The results of descriptive and analytic statistics are reported here.

## Results

### Background characteristics of the CHWs

The vast majority of the CHWs were female (Table 2). Half of the CHWs in the study were between the 31–40 years age group and the majority (67%) had achieved ordinary level education. These characteristics are representative of the typical CHWs in the full Living Goods Uganda program.

**Table 2:** Background characteristics of the CHWs

Characteristic	N	Percent
**Sex (N= 211)**		
Male	7	3%
Female	204	97%

**Age group** **(Years) (N=211)**		
21–30	24	11%
31–40	106	50%
41–50	65	31%
51–60	16	7%

**Highest Education (N=211)**		
Primary	28	13%
O level	142	67%
A level and above	41	19%

### Achievement on health KPIs

On all KPIs, the percentage of CHWs hitting pre-set KPIs is higher among CHWs under peer supervision compared with those under the standard model of supervision (Table 3). Most notably, CHWs under peer supervision outperformed those on the standard supervision model in conducting sick child assessments (85% versus 56% for children under 5 years and 89% versus 65% for children under 1 year). Other KPIs that had significant differences in performance included under-5 sick child treatment, under- 1 sick child treatment, and postnatal care visits within 48 hours of birth. In addition, stock of high-impact items including artemisinin-based combination therapy (ACTs) for malaria treatment, malaria rapid test kits, oral rehydration solutions, zinc for diarrhoea management, and amoxycillin for pneumonia management among CHWs under the peer supervision model was significantly different (0.0394< 0.05) at 92% compared with 63% among CHWs under the standard supervision model. There was also a notable difference in attrition with 10% attrition among CHWs under the peer supervision model versus 17% among non-peer supervised CHWs.

**Table 3:** Comparison of CHWs hitting KPI targets for standard and peer supervision models

KPIs (% hitting target)	Standard model	Peer supervision	P- Value
U5 sick child assessments	56%	85%	0.041
U1 sick child assessments	65%	89%	0.1292
U5 treatments	32%	41%	0.007
U1 treatments	40%	53%	0.000
On-Time-Postnatal follow up	32%	34%	0.02
CHW attrition rate	17%	10%	0.000

**Figure 2:**
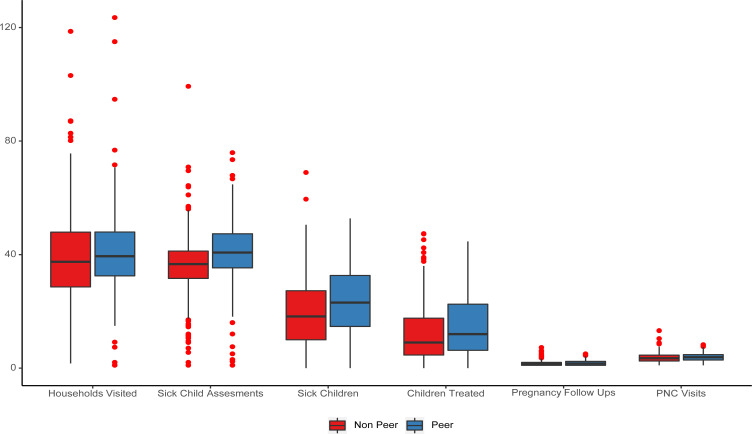
Comparison of CHW performance

Figure 2 shows a comparison of CHW performance aggregated for January to December 2019 for the KPIs monitored under the pilot. Overall, there was significantly better performance among CHWs under peer supervision on the number of households visited, sick children assessed, and sick children identified and treated.

### Participant experiences during the pilot

The CHWs were asked to rate their satisfaction with the peer supervision they had been receiving on a scale of 1 to 5 with 1=Worst, 2=Fair, 3=Good, 4=Very Good 5=Excellent. Of the 29 CHWs who participated in the peer supervision pilot evaluation, 23 (79%) rated their experience as excellent while the remaining six (21%) rated it as very good. The main reasons for this high rating were that Peer Supervisors were more accessible to the CHWs and able to solve their daily challenges in a timelier manner, especially those related to data collection and phone use. In addition, the Peer Supervisors spent more time with the CHWs visiting and providing services to the community members, a concept which was not common with the CHSs.

“*It is good and makes me happy that we meet once every week as a group. Because where you were not working well your fellows remind you. The Peer Supervisor is very near all the time. She encourages us to work moving among us and we go to the community with her*.”- CHW

“*I would not have enough time with the CHS and some months I would not even see him*.”- CHW

“*I give her 5. The peer leader reaches out to me every week on phone and in person to ensure I have worked. This makes me work as I do not want to disappoint her and the whole group*” - CHW. The concept of teamwork and physical company while doing work was found to be very attractive, as it helped with working with non-receptive and difficult households. CHWs also appreciated the peer supervision model because it brought supplies much closer to them as their Peer Supervisors brought them weekly stock. Most importantly, Peer Supervisors felt that they had been empowered as leaders.

According to the CHWs, the Peer Supervisors provided the following advantages to the CHWs: they reinforced CHW knowledge, solved immediate phone issues, supported CHWs to visit unreceptive households, were accessible in real time, provided timely reminders, helped in syncing data onto the servers, and delivered medicines and products on time.

### Cost comparison

The total cost to maintain the program per CHW for 1 year was significantly lower at USD$176 per CHW under peer supervision versus USD$273 among CHWs under the standard supervision model (P-value 0.034). The peer supervision model resulted in a 36% reduction in the supervision and in-service training costs.

## Discussion

Our findings during 1 year of implementation have shown that peer supervision is a feasible and less costly model of supervising CHWs and thus may serve as an effective supervision model for others in Uganda and around the world. The most significant benefit of peer supervision from our study is that it improves ownership of the community health work by the CHWs, encourages teamwork, and increases CHW confidence and commitment which ultimately results in improved performance and reduced attrition. Peer supervision also provides a unique opportunity for the peer and supervisor to talk about issues that emerge because of the peer's own life experiences working in similar situations.

CHWs in the pilot owned peer supervision and determined their leadership by voting for their peer leaders. Working in groups resulted in closer social commitments especially among members of the same peer group due to the frequent contact between CHWs. Similar findings were reported by Ngabo and colleagues^29^. The group visits increased individual CHW credibility in their own designated areas as the communities were able to certify that the CHW is truly a Community Health Worker and not self-imposed.

As elaborated by Tulenko^13^, we were able to observe improved performance and reduced attrition as a result of peer supervision, successes that can be attributed to the increased reinforcement and support that is provided by the peers in this model. Although other authors have not yet reported inactive CHWs returning to work as a result of this model, we found that this was the main driver of reduced attrition in the study.

Our findings on peer supervision are consistent with those reported elsewhere on the impact of supportive supervision on worker performance^31^–^35^. One study^31^,^32^ found a 27% difference in children receiving recommended care among CHWs under peer supervision compared to control areas with routine supervision. Prior research has also established peer supervision to be a beneficial strategy as peers can empathize with each other outside of a hierarchical setting^33^, and more cost effective where traditional supervision is too costly^34^. Just like in our study, peer mentoring is popular with both participants and managers^35^.

The findings from this study are in agreement with the recommendations of the WHO CHW programme guidelines published in 2018. The presence of a second layer of supervisors lowers the supervisor: supervisee ratio, improving the frequency of contact between the supervisor and the Peer Supervisors, as well as between the Peer Supervisors and their CHWs. Further investigation of the application of peer supervision at scale among CHWs may be needed to be able to conclusively identify the drivers of improved performance.

## Limitations

The pilot had two limitations, including: 1) The sample size of supervisors was low, and therefore may have low statistical power. However, the use of over 200 CHWs (about half of the CHWs in the district) resulted in an adequate sample of the CHWs. 2) Potential limited external validity due the study being conducted in only one study district. As such, the results from this pilot should be interpreted as suggestive evidence to the effectiveness of the intervention as we await results from a larger scale program.

## Conclusion

Peer supervision is a feasible and less costly model of supervising CHWs and thus may serve as an effective supervision model for others in Uganda and around the world. The most significant benefit of peer supervision from our study is that it ensures ownership of CHW work by the CHWs, encourages teamwork, and improves CHW confidence which ultimately result in improved performance and reduced attrition. Based on these results, the pilot has been extended to cover 1,026 Living Goods-supported CHWs in Mayuge, Mukono, and parts of Wakiso district. An evaluation of implementation at scale in the three districts will be conducted in 2020.

## Figures and Tables

**Figure 3:**
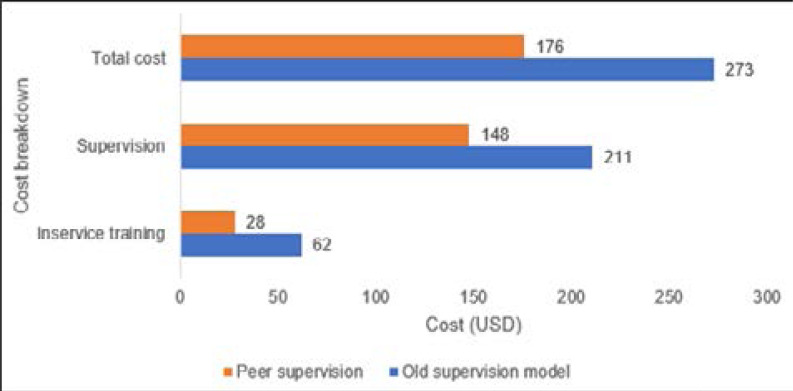
Cost comparison between peer supervision and standard supervision model
